# A Novel Treatment of Schistosomiasis: Nano-Calcium Silicate Incorporating 5% Copper Oxide

**DOI:** 10.34172/apb.2021.004

**Published:** 2020-11-07

**Authors:** Mohamed Fathallah Abou El-Nour, Sayed Hamed Kenawy, Gehan T. El-Bassyouni, Esmat Mahmoud Aly Hamzawy

**Affiliations:** ^1^Department of Zoology, Faculty of Science, Al-Azhar University, Nasr City 11884, Cairo, Egypt.; ^2^Refractories, Ceramics and Building Materials Department, National Research Centre, El-Buhouth St., Dokki, Cairo, 12622, Egypt.; ^3^Imam Mohamed Ibn Saud Islamic University (IMSIU), Collage of Science, Chemistry Dept. Riyadh 11623, Saudi Arabia.; ^4^Glass Research Department, National Research Centre, El-Buhouth St., Dokki, Cairo, 12622, Egypt.

**Keywords:** Anti-schistosomal activity, CS-5%CuO-containing calcium silicate, *S. mansoni*, *S. haematobium*

## Abstract

***Purpose:*** Praziquantel (PZQ) is a well-known drug accredited by the World Health Organization (WHO) for the treatment of schistosomiasis. It shows poor efficiency in patients during the earliest infection phases. Therefore, the search for new alternative drugs was the intention of many researchers.

***Methods:*** In the current study, the effect of different concentrations (ranging from 0.07-10 μg∕mL) of calcium silicate (CS) containing 5% copper oxide [CS-5%CuO] on golden hamster infected by *Schistosoma mansoni* and *Schistosoma haematobium* (Egyptian strains) was evaluated in both in vitro and in vivo. To the best of our knowledge, this is a novel study in investigating the efficiency of CS-5%CuO against both strains of schistosomes. The worms of *S. mansoni* and *S. haematobium* were tested in RPMI-1640 medium in vitro.

***Results:*** The results declare that CS-5% CuO exhibited excellent anti-schistosomal activities on both *in vitro* and in vivo experiments for both Egyptians Schistosoma strains. The most potential effect of the CS-5% CuO was exhibited after 6 h by 10 μg∕mL with significant activity of (*P* value = 0.001).

***Conclusion:*** Therefore, CS-5%CuO may become an innovative treatment for the schistosomiasis.

## Introduction

Schistosomiasis is a highly demolishing tropical disease and is a devastating source of illness in underdeveloped countries.^1^ Bilharziasis is considered a water-born trematodiasis which uses fresh water snails as an intermediate host. There are three main strains infecting humans; *Schistosoma mansoni , Schistosoma haematobium* and *Schistosoma japonicum*.^2^ Such strains are parasitic flukes (trematodes) in the genus *Schistosoma*, found in tropical areas and lead to chronic kidney disease. Schistosomiasis has acute and chronic phases. The acute phase is often short-term and develops in a mild form few weeks following the penetrating of the schistosome parasite through the skin of the host. Unless treated, schistosomiasis may develop into a chronic inflammation which progresses slowly into swelling, fibrosis and necrosis of the affected abdominal viscera.^3^

Mass drug administration in endemic areas using Praziquantel (PZQ) (quinolone derivative) represents a key necessity for schistosomiasis control programs.^4,5^ PZQ is the only drug provided and endorsed by the World Health Organization to treat schistosomiasis.^6,7^ It was used to treat millions of people and it was planned to extend over 235 million people by 2015.^8^ PZQ advantages, is that it has high efficiency on the adult worms of all medicinally significant *Schistosoma* species. In the meantime, its disadvantages are its inefficacy against the early phase of schistosomiasis infection.^9^ In addition, depending on a single drug as the only medicine, even if morbidity is reduced, led to high concerns regarding the evolution of drug resistant schistosomes.^10^ This emphasized the necessity to look for the next generations of anti-schistosomal drugs. Consequently, a great deal of studies has urged the need for new drugs.^11^ *S. mansoni* and *S. haematobium* found primarily across Africa were used to infect the golden hamsters while, *Schistosoma japonicum* found in the Middle East was excluded because, its entire lifecycle would be covered in the vertebrate host.^3^

In nanotechnology the use of nanoparticles (NPs) in medical research is growing rapidly.^12^ Nanomaterials have drawn more attention as anti-parasitic agents. Nanomaterials can be advantageous in both *in vivo* and *in vitro* parasitical studies and applications.^13-15^

Regulating schistosomiasis, is key to discovering a new effective drug. As reported by the National Science Foundation (NSF), nanotechnology deal with the rearrangement of the material dimension between 1-100 nm. Because of their small size, e.g., better solubility, absorption and uptake, nanoparticle-based drugs can reach their required targets more efficiently compared to other drugs.^16^

Nanomedicine, attempts to advance health care.^17,18^ Few researchers summarized the range of intracellular infectious diseases that nanomedicines may be more effective in treating than conventional drugs, such diseases include leishmaniasis and malaria.^19,20^

Taking into account the side effects of antiparasitic drugs and the severity of parasitic diseases, it is important to discover new antiparasitic nanomaterials with low toxicity, low cost and curative effect.^21^ In terms of both chemical and physical properties, CS-5%CuO is low-priced than silver and can be easily mixed with polymers. Recently, CuO nano particles gained importance due to their multifunctional uses in industries and medicine Murthy et al.^22^ Cupric oxide nanoparticles (CuO-NPs) has been of great interest, owing to the attractive physical and chemical properties of these particles. Mabrouk et al decided that the CS-5%CuO showed great cytotoxicity against different cell lines compared with calcium silicates doped with different molar% [1, 3, 7 & 10% CuO].^23^ CS-CuO may also be useful as antimicrobial agents since they can be prepared with high surface areas and unusual crystal morphologies. Consequently, the aim of this research was to assess the efficacy of CS-5% CuO nanoparticles as anti-schistosomal agent in both *in vivo* and*in vitro* scales.

## Materials and Methods

### Material preparation

The present material that was prepared according to the wollastonite composition (CaSiO_3:_ CaO = 48.28g and SiO_2_=51.72g) with doping 5%CuO was added to the solution. The calcium silicate incorporating CuO was prepared via wet method from calcium carbonate (CaCO_3_, El-Gomhouria Company for Trading Chemicals and Medical Appliances, Cairo, Egypt), silica gel (SiO_2_, amorphous, Fluka [Fluka Chemie GmbH, Parent company Sigma-Aldrich, Buchs, Switzerland]) and copper carbonate [Cu_2_CO_3_(OH)_2_, Fluka]. The succeeding reaction can be clarified by the following equations:

$${\text{CaCO}}_{3}+HNO_{3}\overset{Ice�bath}{\to }CaNo_{3}�in�solution$$

CaNO_3_ + amorphous SiO_2_ in solution → CaSiO_3_ (in solution - base) **(1)**

Cu_2_CO_3_(OH)_2_ [calculated as CuO 5%] + HNO_3_ → CuNO_3_ in solution**( 2)**

From **(1)** and **(2)**

$$CaSiO_{3}(base)�in�solution+CuNO_{3�}in�solution\overset{Vigrous�stirring}{\to }CaSiO_{3}/CuO$$

**Figure 1:**
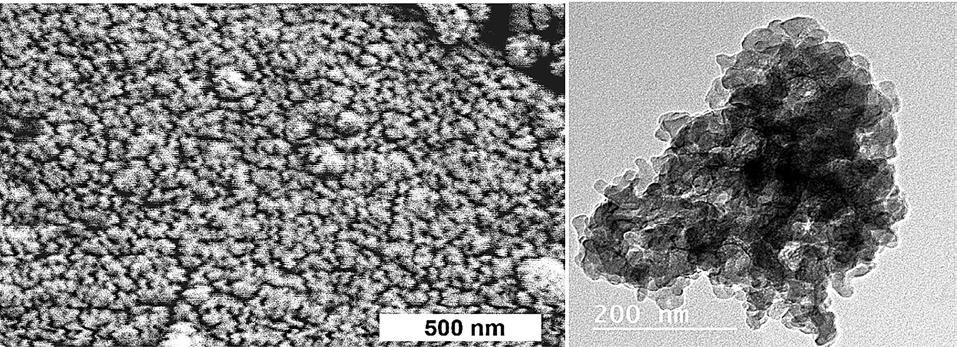
SEM and TEM micrographs of calcium silicate incorporating 5% CuO heat-treated at 550°C/2 h)

The CaSiO_3_/CuO was dried at 100°C, calcined at 550°C for two hours then ball milled into powder. X-ray diffraction analysis (XRD) show the crystallization of quartz [oxide mineral, (SiO_2_)] with little wollastonite (CaSiO_3_), Ca olivine (Ca_2_SiO_4_) and tenorite (CuO). The microstructure (SEM and TEM) revealed the development of nano-particles (Figure 1).^23^

The morphology of the CS-5%CuO was examined via scanning electron microscopy (SEM) (JEOL JXA-840A, Electron Probe Micro-Analyzer, Japan) at 15 kV. Nanoparticles were coated with gold, then examined via SEM apparatus. Particle size of the CS-5%CuO was measured by transmission electron microscopy (TEM; JEOL, Japan, JEM2100, ELECTRON MICROSCPE, TEM-HR). A small quantity of the nanoparticles was dispersed in ethanol, then few milliliters of the solution were dropped onto a copper grid and TEM image was achieved.

### Animal infection by cercarie

The in vivo research protocol followed the guidelines of the National Institutes of Health (NIH) Guide for Care and Use of Laboratory Animals (approval registration No. 16-254). Golden hamster (*Mesocricetus auratus*) were separately housed in plastic cages at room temperature, normal dark/light cycle and leftward to adapt for 5 days. Chow diet and water were provided. Surgical procedure was accomplished under sterile conditions using general anesthesia to suppress both reflex activity and muscle relaxation while losing consciousness. A ratio of ketamine and Bompun (3:1, Agar, Holland) was used for providing such combined effect of anesthesia. The anesthesia was injected intraperitoneally, and its dose was 0.02 mL/30 g hamster body weight. Then the stomach area of the anaesthetized hamster was shaved and ordered on a wooden rack cotton wool dipped in water for moistening the shaven area to permit the easy penetration of the cercarie. About 1 cm metal ring was placed on the shaven area of each hamster, then, a suspension consisting of roughly 250 living cercarie was dispensed in the metal ring using a micropipette at a period of 30 minutes for the cercarie to penetrate into the hamster.^24^

### In vitro study

To culture the parasites, adult worms were washed three times via Roswell Park Memorial Institute (RPMI) 1646 culture medium (Biowhittaker, Lonza, B-4800 Verviers, Belgium). The medium was augmented with L-glutamine, 2090 fetal calf serum, and antibiotics (300 g streptomycin, 300 penicillin, and 160 g gentamycin per mL). Seven couples of worms were moved to each well of the 24 well culture plate (TPP, St. Louis, Mo) containing 1 mL of the same medium. Then l mLof tested material with concentrations (10, 5, 2.5, 1.25, 0.6, 0.3, 0.15 and 0.07 µg∕mL) were added in each well excluding the negative control wells [media with dimethyl sulfoxide,(DMSO)] while, PZQ (10 µg/mL) was used as a positive control. The final size in each well was 2 mL. The plates were incubated at 37°C in a moist atmosphere containing 5% CO_2_ (Thermo Fisher Scientific, Marietta, OH, USA) for 48 hours and monitored at different time intervals (2, 4, 6, 12, 24 and 48 hours). All steps were done in a sterilized laminar flow chamber. The experiment was done in a triplicate and repeated three times. After each incubation time, treated worms were examined for their mating (pairing), motility (worms motor activity changes) and death rate using an inverted optical Olympus

Worms showing no motility were counted as dead. Changes in worm’s motor activity of schistosomes were assessed qualitatively and their motor activity reduction was named as “slight” or “significant” overseeing the adult schistosomes in the *in vitro* experiment at all time intervals.

To observe morphological changes in the tegument of both adult worms, the samples were immediately processed.^25^ The sample was fixed in equal volumes of glutaraldehyde 4% + cacodylate 0.2% for 2 hours. It was then washed in equal volumes of sucrose 0.4% and cacodylate 0.2% for 2 hours. Post fixed in equal volume of osmic acid 2% and cacodylate 0.3% for 1 hour, then washed with distilled water, finally dehydrated in ascending grades of ethyl alcohol for 5 minutes each (30%, 50%, 70% and 90%) then absolute alcohol (100%) for 19 minutes for 3 times. Then examined by Environmental Scanning Electron Microscope (SEM, Inspect S; FEI, Holland) at Electron Microscopy unit of Theodor Bilharz Research institute (TBRI, Embaba, Giza, Egypt).

### In vivo study

#### Liver perfusion

For the *in vitro* bioassay, both*S. mansoni* and *S. haematobium* adult worms of Egyptian strains were brought from victimized infected golden hamsters following the technique of Stirewalt et al from hamster livers after eight weeks in *S. mansoni* and three months in *S. haematobium* of post infection.^26^ Another infected animal group was used for *in vivo* assay.

### Animals 

A total of forty adult golden hamsters of weight 105-130 g were brought from the Schistosome Biological Supply Center (SBSC) at Theodor Bilharz Research Institute (TBRI), Embaba, Giza, Egypt. The animals were divided into four groups. Each group comprised ten animals equally divided into two categories.

Group (1): Negative control contained:

-Non-infected and non-treated hamster

-Infected and non-treated hamster

Group (2): Positive control contained:

- Infected hamster with *S. mansoni* treated with 200mg/kg PZQ (Positive control 1)

- Infected hamster with *S. haematobium* treated with 200 mg/kg PZQ (Positive control 2)

Group (3): Contained:

- Infected hamster with *S. mansoni* treated by CS-5%CuO

- Infected hamster with *S. haematobium* treated by CS-5%CuO

Group (4): Contained:

-Infected hamster with *S. mansoni* treated by calcium silicate without 5%CuO

- Infected hamster with *S. haematobium* treated by calcium silicate without 5%CuO

#### Histopathological assessment

After perfusion and retrieval of the specimens the liver, spleen and kidney were removed from infected treated and non-treated animals. As well as, the healthy organs from the control animals. All organ samples were preserved in 10% formalin for at least 2 weeks. An illustrative portion was brought and washed overnight to get rid of excess formalin. Tissues were dehydrated sequentially in increasing concentrations of alcohols of 50%, 80%, 90% and 96% at hourly stepped intervals.

Tissues were then cleared off alcohol twice into xylene. Infiltration with paraffin wax was accomplished for 3 hours in the paraffin wax oven set at 2°C below the melting point of wax.^27^ Tissues were then emplaced in a fresh molten paraffin wax and left to dry. The tissues were sectioned at 0.7 mm thickness using a microtome, and placed in a hot oven for 15 mins. The tissue sections were de-waxed in xylene, rehydrated and stained with hematoxylin and eosin (H & E) dyes. The stained tissues were slipped with Distyrene plasticizer xylene (DPX), dried and tested microscopically for granulomas.^28^

### The morphology of CS-5%CuO 

Figure 1 signifies the SEM micrograph and TEM image of the calcium silicate incorporating 5% CuO. They demonstrated backed fine rounded grains in the nano-scale size ˂50 nm which confirm the nanosize. The particle size of the prepared materials was found to be reliant on the presence of the CuO, as their obtained particle size was (4.49-6.54 nm) in comparison with that of the pure calcium silicate whose particles size was (19.90-36.23 nm).

### Cytotoxicity

Acute toxicity study was carried out to govern the LD50 of the nanoparticles using a graphical method. Using animal study, signs of toxicity and mortality within 24-72 hours were documented and the LD50 was calculated using the log-probit graph. The LD_50_ of the CS-5%CuO nanoparticles was 365.8760.36 (mg/kg).^23^

Cytotoxicity and cell viability assay was conducted on HFB4 (normal melanocyte). The cell line was obtained from ATCC (American Type Culture Collection). After 24 hours of seeding, the inspected cell line was cultured in a 96 well plate, the medium was changed to serum-free medium containing final concentration of the CS-5%CuO (100 μg/mL) in triplicates. Subsequently, cells were incubated for 2 days, doxorubicin (100 μg/mL) was used as a positive control and 0.5% dimethyl sulfoxide (DMSO) was used as a negative control. Cytotoxicity and cell viability were determined using the MTT (3-(4, 5-dimethylthiazol-2-yl)-2, 5-diphenyltetrazolium bromide) assay. Cytotoxicity results revealed that the investigated nanoparticles are of low efficient on the normal melanocyte (HFB4) above 81.39%, which have been previously described by Mabrouk et al.^23^

### Anti-schistosomal activity 

**Figure 2:**
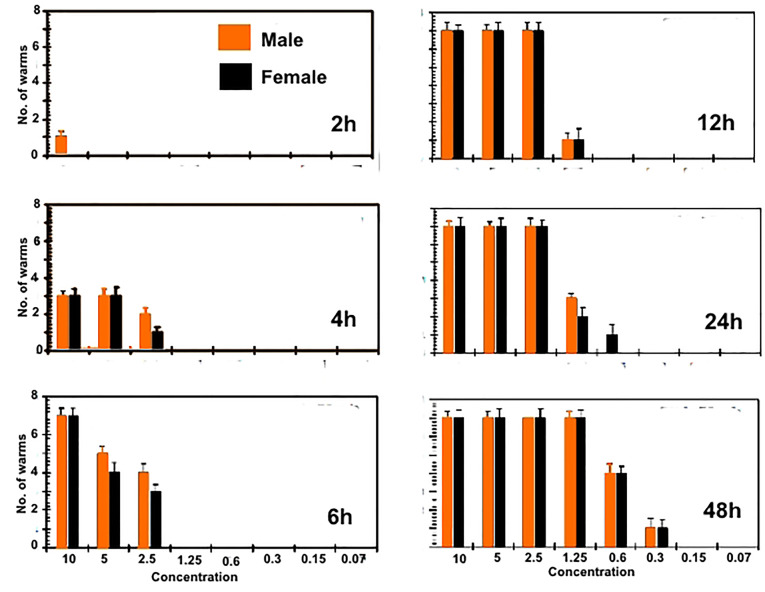
Statistical results of the effect of calcium silicate containing CuO with different concentrations and different times on *S. mansoni*

**Figure 3:**
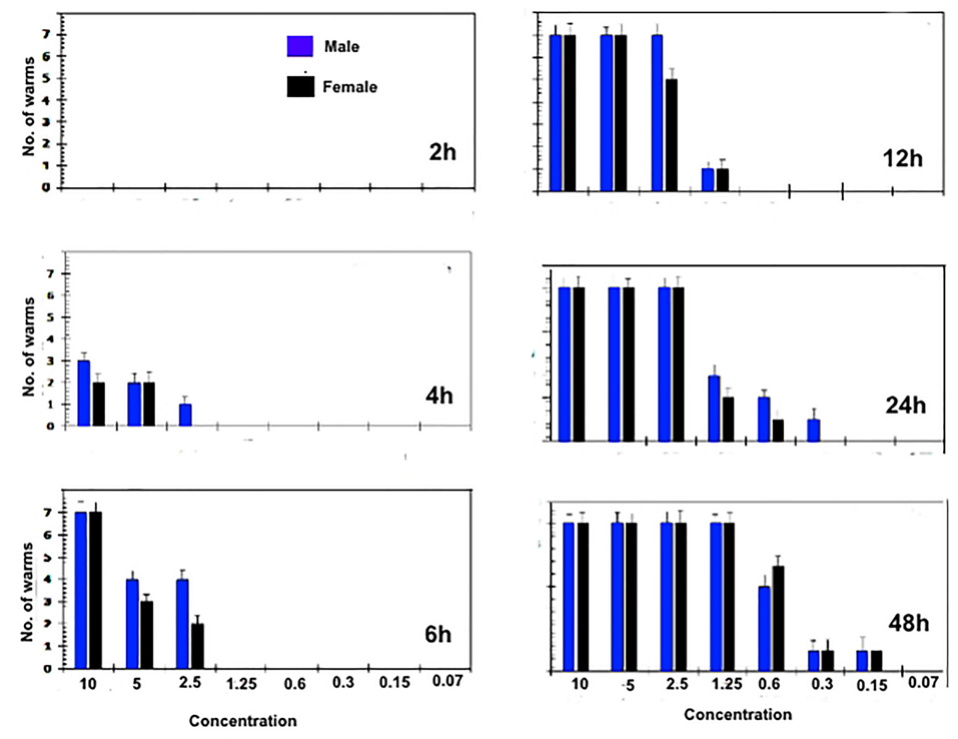
Statistical results of the effect of calcium silicate containing CuO with different concentrations and different times on *S. haematobium.*

The *in vitro* treatment efficacy of CS-5%CuO on adult worms of *S. mansoni* and*S. haematobium* depend greatly on their concentration (10, 5, 2.5, 1.25, 0.6, 0.3, 0.15 and 0.07 µg∕mL). Almost ~95% of the worms had been separated within 2h in both 10, 5 and 2.5 µg∕mL concentrations, although, about 85% of worms had been separated after 4h in both the 1.25 and 0.6 µg∕mL concentrations, and at lower concentrations [i.e. 0.3 & 0.15 µg∕mL] the separation was 60% after 6 hours. In case of concentration (0.07 µg∕mL) the separation of the adult worms was after 12 hours. The known treated control (PZQ 10 µg∕mL) caused separation of adult couple after 4 hours of incubations. However, the efficiency of the lowest concentrations (0.15 and 0.07 µg∕mL) were lower than the higher concentrations as presented in Figures 2 and 3.

During tracing of the parasite motility, a marked decrease in the parasitic movements was noticed in most concentrations. The percentage of worms with reduction in motility was directly proportional to the concentration and to the incubation period. A slight decrease in the motor activity was detected after 2 hours of incubation for all adult worms exposed to 10, 5 and 2.5 (µg∕mL), while, total motility loss happened after 4, 6 and 12 hours, respectively. Other concentrations began to reduce the movement of worms after 12 hours without clear motility loss. Conversely, PZQ (10 µg∕mL) after 2 hours of incubation decreased the motor activity while, after 4h interval, all worms showed complete loss of motor activity. Indeed, the exposure time is a concentration dependent where, at 6 hours CS-5%CuO exposure, both types of worms’ species were 100% destroyed by 10 µg∕mL. Whereas, at concentrations of 5 and 2.5 µg∕mL the time was extended to 12 hours and 24 hours, respectively, for *S. mansoni* (Figure 2), and to 12 h for each of the two previous concentrations against *S. haematobium* (Figure 3). Furthermore, the effect of 1.25 µg∕mL on both worms’ species was similar to a death rate of 100% after 48 h time exposure. Our results delineated that the male worms of both schistosomes were more sensitive against the tested material than the female ones. While, PZQ treated group displayed total death of parasites (100%) after 6h of incubation. Negative control groups were still living at 48 hours of incubation which was considered the end point of the experiment. At 1.25 µg∕mL concentration, the anti-schistosomal effect began to appear after 12 hours of incubation, and the effect was time dependent (Figures 2 and 3). At lower concentration of 0.6 µg∕mL, the effect of the substance began to appear after 24 and 48 hours, while at concentration 0.3 µg∕mL, the effect of the CS-5%CuO began to appear after 48 hours with moderate activity. In case of concentration 0.07 µg∕mL, no anti-schistosomal effect was recorded.

**Figure 4:**
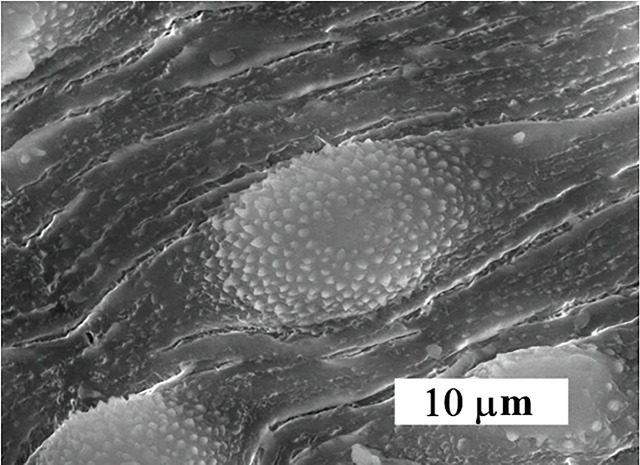
Normal Schistosoma from the hamster tissue

SEM examination from non-treated golden hamster revealed that, the oral sucker in both *Schistosoma* were oval-shape, covered with sharp spines varying in size. But, for the ventral sucker they were rounded, covered with the spine. The ventral surface behind the ventral sucker increases in width and folds ventrally to form the gynaecophoric canal. The dorsal surface of worms was provided with numerous large tubercles bearing spines. The zones between the tubercles were lacking spines. The ventral side of the worm was provided with rows of minute spines. The tegument surrounding the tubercles was wrinkled (Figure 4).

**Figure 5:**
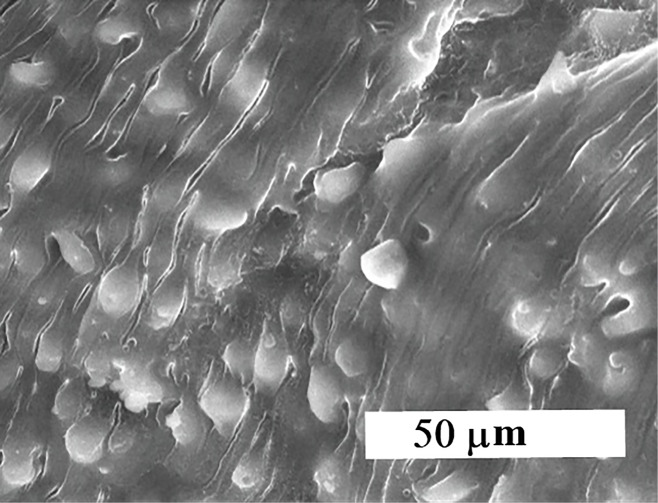
Effect of traditional treatment by PZQ drug.

Clear tegmental changes of*S. mansoni* adult worms in the golden hamster appeared after treatment with CS-5%CuO. Similarly, ultra-morphological variations were observed in both males and females adult schistosomas after 48h incubation *in vitro* with concentrations 10%, 5%, 2.5%, 1.25%, 0.6%, 0.3%, 0.15% and 0.07% from CS-5%CuO. In comparison with untreated parasite there was no any change (Figure 3), tegmental morphological alterations clear for the parasites exposed to CS-5%CuO with variation in the effect relative to the dose ratio of CS-5%CuO. On the other side, the known treatment by PZQ revealed similar tegmental alteration in 100% of *Schistosoma* worms (Figure 5).

### Treatment of S. mansoni and S. haematobium

**Figure 6:**
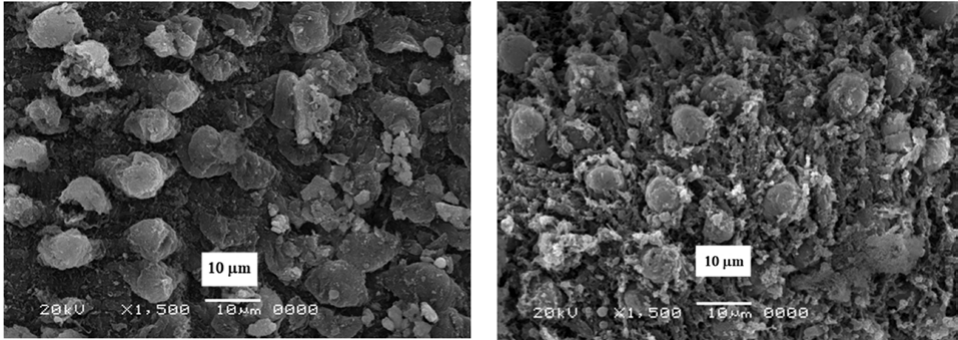
Surface of *Schistosoma mansoni* after treatment by CS-5%CuO.

**Figure 7:**
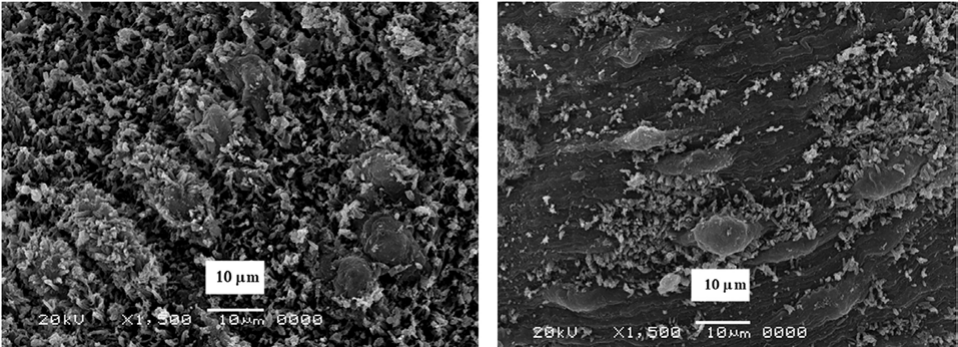
Surface of *Schistosoma haematobium* after treatment by CS-5%CuO.

The morphological changes were clear in the male of both *S. mansoni* and *S. haematobium* since it showed abnormalities in the tubercles, and spine damage (destruction, peeling of spines, tubercles, and tegument peeling or sloughing) specifically on its dorsal surface. The manifestation of bubbles surrounding the morphologically altered tubercles was observed as well as sucker alteration or destruction. The oral sucker of some worms was distorted. While in females the tegument scaling, wrinkling, and erosion (contraction and peeling of dorsal region) and suckers’ alterations or destruction were noticed (Figures 6 and 7).

### Histopathological assessment 

**Figure 8:**
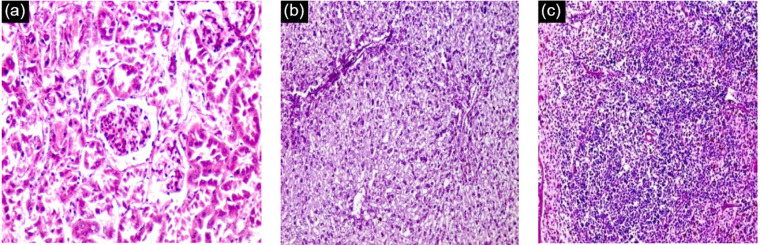
Histological section of negative control (Healthy control) staining with H & E: a) Control kidney showing normal cortical structure (X200). b) Control liver showing normal hepatic lobular architecture (X100). c) Control Spleen showing normal architecture (X100).

Histological examination of kidney, liver and spleen sectors of control group (healthy group) using light microscopy have been established. It revealed that, the cortical parenchyma of the kidney contains a number of renal corpuscles together with proximal and distal renal tubules (X200) (Figure 8). In addition, liver hepatocytes extended from the central vein to the periphery of the hepatic lobules and the portal tract was shown at X100 (Figure 6). However, the spleen presented with a normal architecture, consisted of white and red pulps enveloped by a capsule of dense connective tissue. The white pulp consisted of a central, T-cell rich zone, and a periarterial lymphoid sheath enclosed by B-cell-rich primary follicles. The marginal sinus disconnected the white pulp from the red pulp. Marginal sinus was embedded in a layer of marginal zone lymphocytes (X100) (Figure 8).

**Figure 9:**
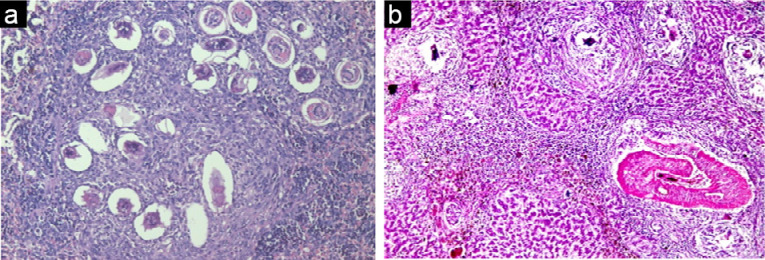
Histological section of infected untreated control staining with H & E: a) Spleen revealed aggregate of deposited bilharzia ova, surrounded by lympho-epithelioid tissue reaction (X200). b) Liver showing multiple egg and a worm impacted inside a portal vein (X100).

However, the effect of the parasite on infected untreated control group, showed no obvious effect on the kidney, nevertheless there was an effect on both the liver and the spleen that revealed pathological chronic granulomatous lesions in the hepatic parenchyma. Such lesions were formed of several bilharzial eggs containing miracidia, enclosed by abundant chronic inflammatory cells in the form of epithelioid cells, lymphocytes, plasma cells, macrophages, and eosinophils forming granuloma accompanied with severe fibrosis (Figure 9). Also, the spleen showed ova surrounded by inflammatory cellular reaction and the borders in between white and red pulp began to fade. Roughly splenic cells were evaluated. Most advanced cells were darkly stained with big sinusoidal spaces.

**Figure 10:**
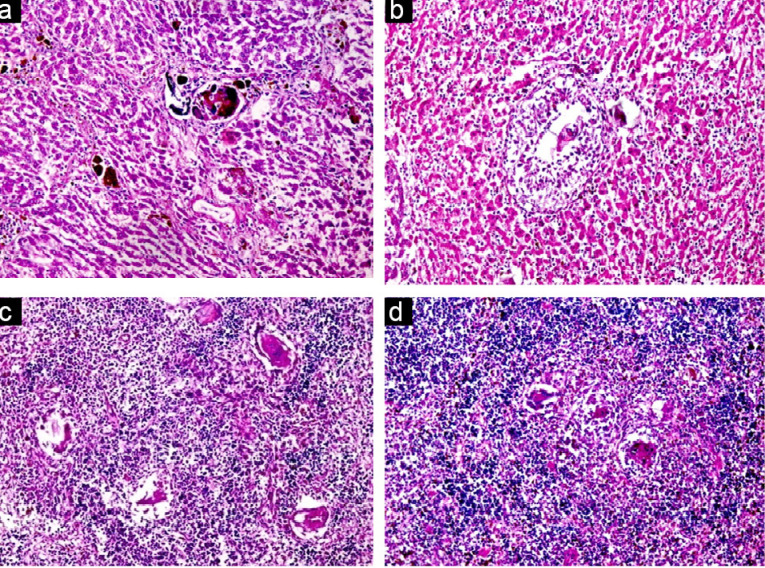
Histological section of infected treated group staining with H & E: a) Liver showing calcified SH ova, mostly engulfed by multinucleated giant cells, with little disturbance of hepatic lobular architecture (X200). b) Liver showing epithelioid egg granuloma (X200). c) Spleen showing many fresh deposited and degenerated SH ova (X200). d) Spleen showing some degenerated ova, surrounded by lympho-epithelioid cellular inflammatory cellular infiltration (X200).

Histological liver sections treated with CS-5%CuO exhibited diffuse infiltration of liver parenchyma via chronic inflammatory cells devoid of observed eggs or fibrosis regions (Figure 10). Absence of bilharzial eggs and fibrosis with substantial reduction of liver parenchyma infiltration by the chronic inflammatory cells was revealed. On the other hand, spleen sections exhibited more or less degeneration of ova, surrounded by lympho-epithelioid cellular inflammatory cellular infiltration.

## Discussion

In tropical regions, schistosomiasis is a parasitic disease that affects human health. World Health Organization (WHO) provided a program for studying the development of new anti-parasitic drugs and encourages worldwide synthesis of new compounds for these parasitic diseases.^29^ Previous studies exhibited the effectiveness of using nano particles of calcium silicate CS-CuO to fight bacteria and fungus. Clusters containing 5% CuO showed inhibition zone diameter (IZD) in the range of 30 and 22 mm, respectively. Other clusters containing 3% CuO showed IZD of 20 mm. Clusters of wollastonite with/without CuO prepared via wet precipitation route can be considered as a novel antimicrobial agent for bone implantation.^30^ In contrast, calcium silicate incorporating CuO effectively motivated bone regeneration. Also, it was postulated that the CS-CuO materials is recommended for treatment of cancer. The upcoming triumph of copper incorporation into compounds in the clinic demands made an link between biomedical scientists, clinicians and likewise chemists.^31^ In the present study, different concentrations of CS-5%CuO against *S. mansoni* and*S. haematobium* (Egyptian strains) were experimentally assessed *in vitro* and in vivo in infected golden hamster. To the best of our knowledge, this study may be the first investigating the efficacy of CS-5%CuO against Egyptian *Schistosoma* strains. Our results revealed that, CS-5%CuO especially at concentration 10, 5 and 2.5 (µg/mL) showed strong anti-schistosomal activity against male and female of *S. mansoni* and*S. haematobium.* Nanomaterials play a vital role in the treatment of many parasitic diseases.^32^ Dkhil et al examined the influence of selenium nanoparticles (Se-NPs) on mice infected by schistosome. Se-NPs ameliorated the hepatic histopathology and reduced the granulomas diameters. Furthermore, this treatment increased the glutathione level while; the levels of nitrite/nitrate and malondialdehyde were significantly decreased. Dkhil et al, in 2017 explained, the role of gold nanoparticles (Au-NPs) in contradiction of splenic injury in mice infected with S. mansoni. The treatment of mice with gold NPs reduced the extent of histological impairment and oxidative stress in the spleen tissue.^33^ Lately, Dkhil et al concluded the curative effects of NPs related to their antioxidant activities; consequently, they proved their anti-schistosomal activities in mice.^34^ Oliveira et al proposed different strategy to prompt protection against *S. mansoni*  infection by creating a vaccine founded on chitosan nanoparticles together with the antigen SmRho coated with alginate.^35^ It was capable of modulating the granuloma area that signifies the major pathological response in schistosomiasis and also to encourage the defense against infection *S. mansoni*. Our results are matching with other studies which inspected the effect of anti-schistosomal drug against tegument of schistosomes.^36^ The changes caused by CS-5%CuO was more noticeable in male tegument than in that of females. These findings were in agreement with the findings of Staudt et al.^37^ This may be elucidated by the point that most of the female’s body is walled in the gynaecophoric duct of the male [i.e., not in direct contact with the host’s microenvironment].^38^

The alteration in the surface architecture of *S. mansoni* and *S. haematobium* worms as a result of treatment with CS-5%CuO were more or less like the tegmental alterations in *Schistosoma mekongi* worms sheltered in mice treated with artesunate (C_19_H_28_O_8_) as observed by Jiraungkoorskul et al^39^ or that studied by Staudt et al on the effects of the enantiomers of PZQ and its main metabolite on in vitro*S. mansoni.*^37^ The current results revealed that the morphological alterations could be a mechanism for killing the worms using CS-CuO. Strong damage to the suckers led to the loss of the parasite ability to stick to the blood vessels and rendered the difficulty of ingestion of nutrients from the blood. Furthermore, the damage to the worm’s body tegument would destroy the defense system of the worm, so that it could easily be attacked by immune system of the host.^40^ On the other hand, the *in vivo* anti-schistosomal activity of CS-CuO on both strains of infected hamster was evaluated concerning histopathological changes in liver, spleen and kidney. The results showed that, both liver and spleen tissues showed strong damage in infected untreated control, but kidney was not affected. This affection may be attributed to the demolition in the hepatic tissue leading to the incapability of the liver to metabolize proteins and fats, or else to consume glucose and store glycogen.^41^ On the contrary, CS-5%CuO treated groups showed marked improvement on both liver and spleen tissues. Our results are in agreement with other researchers.^42^ Histopathology of the liver and spleen of the CS-CuO cured hamster shown fewer granulomas than in non-treated group. Other results by Wynn and Cheever, 2007 stated that the reduction of granuloma diameter might be attributed to the reduction of type III procollagen which is in charge of the granuloma’s creation.^43^ We could also clarify that, the reduction of granuloma size by CS-CuO may be owing to the suppression of the T helper (TH) cell differentiation (Th1 and Th2) lymphocytes and their cytokines. Such suppression may facilitate the formation and development of granuloma.

Khalil et al studied the effect of iron nanoparticles on adult worms of *Schistosoma mansoni* using different concentrations of 30 and 60 mg/L. Their results exhibited activity on worms, and that the mortality rate in the concentration of 30 mg/L was 15%, 20% and 100% after incubation period of 2, 3 and 12 hours, respectively. While for the concentration of 60 mg/L, it recorded 55%, 65%, 77% and 100% after incubation period of 1, 2, 3 and 48 hours, respectively.^44^ In 2012, Luz et al assessed the in vitro anti-schistosomal activity of curcumin combined with nanoparticles of poly (lactic glycolic acid) (PLGA) with a 100% mortality rate at 50 and 100 50μM after 12 and 24 hours of incubation, respectively. It led to a decrease in motor activity after 12 hours of incubation at 40μM and 30μM. Besides, at concentrations 40μM of the curcumin loaded with PLGA nanoparticles caused partial changes in adult integument, presence of alteration and structural vesicles after 48 hours of incubation.^45^ As a result, CS-5%CuO may become a pioneering treatment for schistosomiasis.

## Conclusion

The results declare that CS-5%CuO exhibited excellent anti-schistosomal activities on both in vitro and in vivo experiments for both strains of Egyptian schistosomes. The most potential effect of CS-5%CuO was exhibited after 6 hours by 10 µg∕mL with significant activity of (*P* value = 0.001). Consequently, calcium silicate incorporating CuO may become a novel talented candidate for the development of new schistosomiasis treatments.

## Conflict of Interest

Authors confirm that there are no conflicts of interest.

## Ethical Issues

The in vivo research protocol was reviewed and approved by the Animal Care Committee of the National Research Centre, Egypt, which follows the guidelines of the National Institutes of Health (NIH) Guide for Care and Use of Laboratory Animals (approval registration No. 16-254).
